# Disease Pattern May Explain Angina After Chronic Total Occlusion Revascularization

**DOI:** 10.1016/j.jacadv.2026.103236

**Published:** 2026-09-11

**Authors:** Shigetaka Kageyama

**Affiliations:** Department of Cardiology, Shizuoka City Shizuoka Hospital, Shizuoka, Japan

Daoulah et al^1^ should be congratulated for the Gulf Chronic Total Occlusion (CTO) Registry. Coronary artery bypass grafting (CABG) was associated with the lowest angina burden at follow-up. The authors appropriately acknowledge that missing comparable baseline angina grades precludes attributing symptom improvement to treatment. We suggest that coronary disease pattern may nevertheless help explain this intriguing signal.

Collet et al^2^ found residual angina after percutaneous coronary intervention (PCI) in approximately 40% overall, but in 52% with diffuse vs 27% with focal disease. Our prior editorial emphasized that symptoms after technically successful PCI may reflect both residual diffuse epicardial disease and microvascular dysfunction.^3^

This framework is relevant because 71.1% of CABG patients had three-vessel disease, with a median of 3 grafts and the CTO vessel grafted in 98.6% of those with documented status.^1^ The 98.0% adjusted prevalence of Canadian Cardiovascular Society class I may therefore be biologically plausible. PCI generally treats dominant focal gradients, whereas CABG can deliver flow distal to multiple diseased epicardial segments across several territories. Diffuseness may thus modify symptomatic effectiveness of PCI vs CABG.

Conversely, even anatomically complete CABG cannot normalize an elevated index of microvascular resistance, endothelial dysfunction, or vasomotor abnormalities; residual angina may persist despite complete epicardial revascularization. Future CTO studies should combine baseline and follow-up patient-reported outcomes with focal-versus-diffuse physiology, revascularization completeness, and postrevascularization epicardial and microvascular assessment. This could turn the Gulf CTO observation into a phenotype-based treatment hypothesis.
